# A Polydopamine-Based Molecularly Imprinted Electrochemical Sensor for Fentanyl Determination

**DOI:** 10.1021/acsomega.5c06732

**Published:** 2025-08-12

**Authors:** Michelle Tong, Rajesh G. Pillai, Alexander Kobryn, Zhimin Yan, Nora W. C. Chan, Abebaw B. Jemere

**Affiliations:** † National Research Council Canada - Quantum and Nanotechnologies Research Centre, Edmonton, Alberta T6G 2M9, Canada; ‡ 194375 Defence Research and Development Canada—Suffield Research Centre, Medicine Hat, Alberta T1A 8K6, Canada; § Department of Chemistry, Queen’s University, Kingston, Ontario K7L 3N6, Canada

## Abstract

A molecularly imprinted polymer (MIP)-based electrochemical sensor for the rapid detection of fentanyl is reported. The sensor was prepared by electrochemically grafting polydopamine on a carbon nanofiber–Pt nanoparticle composite-modified screen-printed electrode. Dopamine was identified as a suitable functional monomer via in-silico modeling and was electropolymerized via cyclic voltammetry in the presence of fentanyl to form the MIP sensor. The properties and morphology of the sensing material were characterized with spectroscopy, microscopy, and electrochemical techniques. Factors influencing the sensor performance were studied and optimized. Under optimized conditions, the MIP sensor response followed the Langmuir–Freundlich binding isotherm with a dissociation constant (*k*_d_) of 16.13 μM and a limit of detection of 0.094 μM fentanyl. The sensor displayed good run-to-run repeatability and batch-to-batch performance reproducibility with relative standard deviations of 6.7% (*n* = 5) and 9.1% (*n* = 3), respectively. Three sensors, prepared and tested in parallel, showed excellent storage stability in a fridge under a humidified environment for 4 weeks with relative standard deviations of ≤10%. The developed MIP sensor presented suitable selectivity when interrogated with solutions composed of equimolar concentrations of fentanyl and glucose, acetaminophen, theophylline, morphine, naloxone, codeine, or norfentanyl. The sensor was also successfully tested in artificial urine samples, indicating that it is a promising candidate as a rapid testing method in fentanyl investigation.

## Graphical abstract

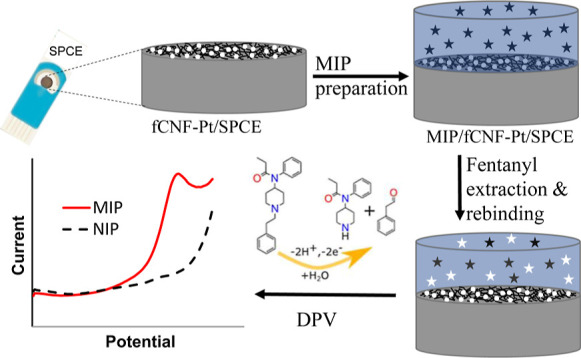

## 1 Introduction

Fentanyl, C_22_H_28_N_2_O, is a monocarboxylic acid amide commonly prescribed to manage severe pains during surgery and due to advanced cancer.^1^ However, the drug and its structural analogues are increasingly being used illicitly, causing the ongoing opioid crises globally and posing real risk to society worldwide.^2^ For example, a 2024 data from the US CDC shows more than 85,000 Americans died due to opioid overdose, the majority of which due to accidental fentanyl overdose.^3^ Canada has also seen a considerable rise in fentanyl-related deaths in recent years, with ∼81% of all opioid-related deaths in 2024 caused by fentanyl overdose.^4^ Addressing the fentanyl epidemic crises needs a holistic approach, including improved detection platforms to abet first responders and law enforcement officials obtain valuable information diagnosing nonresponsive patients and to detect fentanyl-contaminated contrabands. Current analytical methods for the detection of fentanyl are primarily based on chromatography techniques coupled with mass spectrometry or UV readouts,^5–8^ immunosensing,^9,10^ and surface-enhanced Raman spectroscopy.^11^ Although these techniques provide accurate results, they require complex protocols, highly trained personnel, and large and expensive instrumentation, making them restricted to centralized laboratory analysis. Field-deployable fentanyl test strips and desktop Fourier-Transform Infrared Spectrometer (FTIR) are being used for on-site monitoring of fentanyl; however, these techniques are prone to error (high degree of false negative and false positive) and require fairly high concentration.^12^ Thus, fast responding, easy to operate, inexpensive, and portable fentanyl detection platforms with high selectivity, sensitivity, and low detection limits that can be deployed in a large scale for infield screening would be highly beneficial in attempts to combat the opioid epidemic.^13^ Electroanalytical methods exhibit the aforementioned advantages^14–16^ and are considered good candidates for the development of fieldable fentanyl sensors.^17,18^ In recent years, a number of electrochemical fentanyl sensors are reported in the literature using various carbon,^19–29^ NiO,^18^ and Zn­(ii)- and ZIF-8-metal–organic framework (MOF)-modified electrodes.^30,31^ These reports, however, relied on the direct adsorption of fentanyl onto the electrodes’ surfaces, without a specific receptor and suffering from poor selectivity.^13,32^

Electrochemical sensors utilizing molecularly imprinted polymers (MIPs) as recognition elements have attracted great attention in chemical and biological sensors’ development due to their high selectivity, low cost, ease of preparation, good physical and chemical stability, and reusability.^33–36^ Molecular imprinting, which involves polymerizing functional monomer(s) in the presence of a template molecule (usually the target analyte or a molecule of similar chemical functionality and size), is a well-known and cost-effective method for preparing selective binding sites in synthetic polymers using molecular templates and functional monomers.^34^ Removal of template molecules from the polymer network generates vacant binding cavities which are complementary in size, shape, and functional groups to the target analyte. The binding sites formed in the cavities result in an affinity and selectivity toward target analytes, mimicking a natural bioreceptor in an antigen–antibody kind of relationship. To date, there exists only one literature report demonstrating the development of an MIP-based fentanyl electrochemical sensor, where the authors used l-arginine, an amino acid, as a functional monomer in the preparation of electropolymerized MIP on a graphene oxide-modified glassy carbon electrode using a wide potential window.^32^ Electropolymerization of MIPs allows the growth of a uniform polymer layer directly onto the electrode surface and to easily manipulate parameters such as polymer thickness, morphology, and topography for each template by varying charge passed during polymerization.^37^ However, when imprinting electroactive analytes such as fentanyl using electropolymerizable functional monomers, the polymerization potential window must be carefully selected to maintain the structural rigidity of the template molecules. Polymerization in potentials that would cause oxidation or reduction of the template molecules will result in template degradation, leading to poor imprinting and loss of specificity and performance of the resulting MIP.^37^ Here, we used dopamine to form a fentanyl MIP on a carbon nanofiber–platinum nanoparticle composite-modified screen-printed carbon electrode (fCNF-Pt/SPCE). Dopamine, unlike l-arginine, can be polymerized in a potential window that does not oxide fentanyl. In this work, the selection of dopamine as the functional monomer was guided by in silico modeling, using Density-Function Theory. Dopamine, with its catechol and aminoethyl side chain, offers functional groups that can undergo noncovalent interactions such as hydrogen bonding and π–π interactions with the template (fentanyl) molecules to form a highly selective MIP. Polydopamine-based imprinted surfaces provide well-defined and stable cavities, which is vital for the efficiency and reproducibility of MIP sensors. In the literature, MIPs of polydopamine have been prepared via bulk polymerization and electropolymerization techniques on various substrates^38^ and applied for sensitive detection of a number of small molecules,^39–41^ proteins,^42,43^ bacteria,^44,45^ and viruses.^46,47^

In this work, we developed a polydopamine MIP electrochemical sensor for the rapid and selective determination of fentanyl. Fentanyl-imprinted polymers (MIPs) were prepared on carboxylic acid-functionalized carbon nanofiber (fCNF)–Pt nanoparticle composite-modified screen-printed carbon electrode (fCNF-Pt/SPCE) surfaces. The use of SPCEs in electrochemical sensor fabrication has received significant attention in recent years due to their low cost, ease of mass production, ability to customize their surfaces, disposability, and amenability to miniaturization and integration into portable devices.^48^ In a recent report, our group demonstrated a direct electrochemical oxidation of fentanyl on fCNF/SPCEs, achieving good analytical performance.^27^ The sensor, however, suffered poor selectivity due to the absence of a selective recognition moiety. In this report, we prepared a polydopamine-based fentanyl MIP sensor on fCNF-Pt/SPCEs using cyclic voltammetry (CV) to improve selectivity. The resulting sensor exhibited large numbers of fentanyl recognition sites with significant binding affinity (micromolar dissociation constant), good performance, and fabrication reproducibility and stability over time. The sensor rendered high selectivity when tested against structurally similar compounds (morphine, codeine, naloxone, and norfentanyl) and common street drug-cutting agents (glucose, theophylline, and acetaminophen). The sensor was also successfully tested to quantify fentanyl in synthetic urine samples.

## 2 Experimental Section

### 2.1 Chemicals and Materials

One mg/mL Cerilliant-certified reference methanolic solutions of fentanyl (FEN), morphine (MO), codeine (CO), and norfentanyl (NOR), as well as naloxone hydrochloride dihydrate, glucose, acetaminophen, theophylline, conical carbon nanofiber (CNF), sodium phosphate dibasic, sodium phosphate monobasic, sulfuric acid, nitric acid, ethanol, potassium ferrocyanide, potassium ferricyanide, potassium nitrate, and dopamine hydrochloride, were purchased from Sigma-Aldrich Canada (Oakville, ON). Artificial urine was purchased from Pickering Laboratories (Mount View, CA, USA). SPCEs consisting of a 12.6 mm^2^ C working electrode, a Ag reference electrode, and a Pt auxiliary electrode were acquired from Metrohm DropSens (DPR-150, Oviedo, Spain). Deionized (DI) water with a resistivity of 18 MΩ·cm (Millipore Canada, Mississauga, ON) was used for solution preparation and electrode rinsing.

Individual aqueous stock solutions of FEN, MO, CO, and NOR were prepared by first evaporating the bulk methanol solvent using a gentle N_2_ stream in a fume hood at room temperature overnight, followed by a 30 min high-vacuum drying to further remove trace solvent. Complete removal of methanol was required as it exhibited similar oxidation potential to FEN. Individual aqueous stock solutions of FEN, MO, CO, and NOR were then prepared by resuspending the dried chemicals in DI water and stored at 4 °C until use. Daily working solutions were prepared in 0.1 M phosphate buffer (PB, pH 8.0), except during the pH optimization study.

### 2.2 MIP Sensor Fabrication

Scheme 1 shows the stepwise fabrication of the MIP sensor. Prior to modifying SPCE, carbon nanofibers were first functionalized with –COOH groups as previously described,^27^ to promote their dispersibility and stability in aqueous solutions. Briefly, 1 g of CNF was added to a 40 mL mixture of HNO_3_/H_2_SO_4_ (1:3 v/v) and heated to 50 °C under magnetic stirring for 8 h. The resultant mixture was cooled to room temperature, centrifuged, and washed with DI water until neutral pH was obtained. The product was dried in a freeze-dryer for 3 days, resulting in –COOH-functionalized CNFs (fCNFs). Zeta potential (Zetasizer, Malvern Panalytical, St. Laurent, QC, Canada), Fourier transform infrared (FTIR, Agilent Technologies, Penang, Malaysia), and Raman spectra (DXR2, Thermo Scientific, Toronto, ON, Canada) of as-received and fCNFs were measured and compared.

**Scheme 1:**
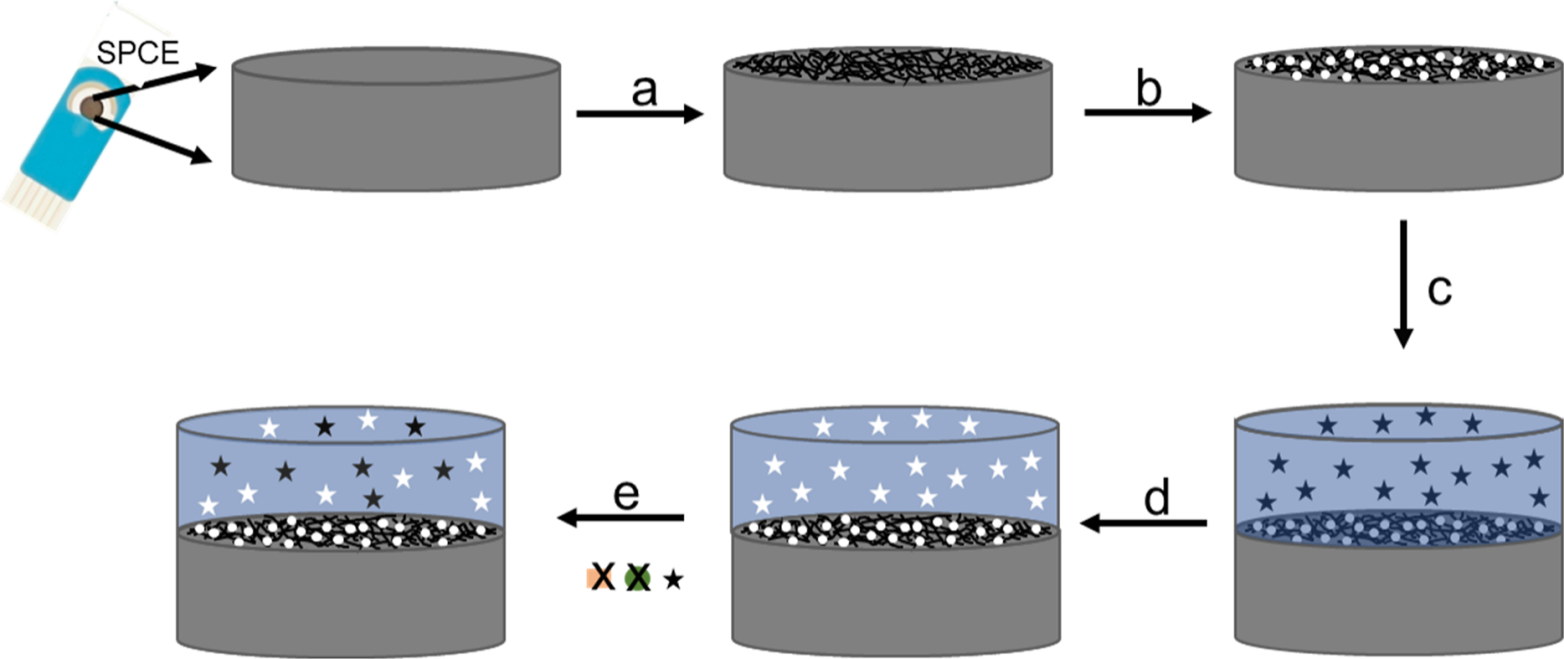
Graphical Representation of the Fabrication of a MIP-Based Fentanyl Sensor: (a) Deposition of Functionalized Carbon Nanofibers on SPCE, (b) Electrodeposition of Platinum Nanoparticles, (c) Electropolymerization of Dopamine-Fentanyl MIP, (d) Fentanyl Extraction, (e) Fentanyl Rebinding and Direct Detection. Tiny white dots, dark spaghetti lines, stars, orange squares, and green circles represent Pt nanoparticles, carbon nanofibers, fentanyl, and other tested compounds, respectively

Prior to modification with fCNF, a bare SPCE was rinsed with DI water, dried with a stream of N_2_, and activated with 0.1 M H_2_SO_4_ by cycling between −0.2 and +1.3 V for 5 cycles until a stable CV curve was obtained. Then, 8 μL of an ethanolic suspension of 1 mg/mL fCNF was pipetted onto the SPCE and dried at room temperature for 30 min, resulting in fCNF/SPCE. The fCNF/SPCE was further modified with platinum nanoparticles via electrochemical reduction of a 1 mM chloroplatinic acid in 0.1 M H_2_SO_4_ solution using chronoamperometry at an applied potential of −0.5 V for 90 s, resulting in fCNF-Pt/SPCE. After each modification step, the electrode was thoroughly washed with DI water and dried under a N_2_ stream.

To fabricate the MIP sensor on the fCNF-Pt/SPCE surface, a prepolymerization mixture consisting of 2.1 mM dopamine and 0.35 mM fentanyl was prepared in 0.1 M PB (pH 8), purged with N_2_ for 15 min, and incubated for 1 h in the dark. Then, 100 μL of the prepolymerization mixture was pipetted onto the modified electrode and electropolymerized via CV in the potential window of −0.5 and +0.5 V for 10 cycles at a scan rate of 50 mV s^–1^, resulting in MIP/fCNF-Pt/SPCE. A control nonimprinted polymer (NIP) was fabricated on fCNF-Pt/SPCE under the same experimental conditions as the MIP but without fentanyl in the prepolymerization mixture. Following electropolymerization, the MIP/fCNF-Pt/SPCE and NIP/fCNF-Pt/SPCE were washed with DI water and dried with a gentle N_2_ stream. Extraction of imprinted fentanyl molecules was obtained using CV in a potential window of −0.2 to +0.85 V for 5 cycles at a scan rate of 50 mV s^–1^ using 0.1 M PB (pH 8) as an electrolyte. To obtain optimal sensor performance, sensor fabrication parameters including the monomer-to-template ratio, number of CV polymerization cycles, and polymerization pH were optimized. Long-term storage stability of three MIP sensors, prepared and processed in parallel, was investigated by monitoring the differential pulse voltammetry (DPV) responses of the sensors following incubation with 5 μM fentanyl prepared in 0.1 M PB, pH 8.0. Following every DPV measurement, the sensors were regenerated via CV in 0.1 M PB, pH 8.0, for 5 cycles for repeated use. When not in use, the sensors were washed and stored in water under a humidified environment at 4 °C. Energy-dispersion spectroscopy (EDS, Hitachi S-5500, Japan) analysis of the modified electrodes, including that of the as-prepared MIP/fCNF-Pt/SPCE and after repeated use, was performed and compared.

### 2.3 Instruments

Electrochemical measurements were performed using 100 μL solutions pipetted onto the three-electrode sensor system, with the MIP/fCNF-Pt/SPCE as a working electrode and a PalmSens potentiostat controlled by PSTrace 5 software (Utrecht, The Netherlands, www.palmsens.com). One mM K_3_[Fe­(CN)_6_/K_4_[Fe­(CN)_6_], prepared in 0.1 M KNO_3_, was used as the redox probe for all electrochemical characterizations of the sensor fabrication steps. DPV experiments were performed by scanning the potential from 0.2 to 0.85 V using a step potential of 10 mV, pulse amplitude of 50 mV, pulse period of 50 ms, and a scan rate of 100 mV s^−1^ at room temperature. Electrochemical impedance spectroscopy (EIS) was performed in a frequency of 0.1 Hz to 100 kHz using an AC amplitude of 10 mV at an open-circuit potential. Morphologies of the modified electrodes were also characterized by scanning electron microscopy (SEM, Hitachi S-5500, Japan, www.hitachi.com).

### 2.4 Computational Selection of the Monomer

Five laboratory available and commonly used monomers (2-hydroxyethyl methacrylate, 4-aminobenzoic acid, 4-vinylbenzoic acid, dopamine, and methacrylic acid) were studied for their ability to form a stable complex with the target fentanyl using in silico modeling. Density function theory (DFT) computations were carried out by ORCA—Version 5.0.^49^ The molecular geometries of the template, functional monomers, and monomer–templet complex were optimized with ratios of 1:1 to 1:6 and visualized with the Biovia Discovery Studio Visualizer.^50^ Theoretical calculations were carried out using Becke ’88 exchange and Perdew ’86 correlation density functional, valence double-ζ basis set with ″new″ polarization functions, and atom-pairwise dispersion correction to the DFT energy with Becke–Johnson damping. The initial geometry and other parameters for the template and functional monomers were taken from PubChem. To approximate the four-index electron repulsion integrals by two- and three-index tensors, we used the density fitting option, also called the resolution identity, and Weigend’s auxiliary basis set for Coulomb fitting. In the calculations of binding energies, the below equation was used:

$$\Delta E=E_{complex}-E_{template}-\sum E_{monomer}$$

where *E*_complex_ is the total energy of the fentanyl–monomer complex, *E*_template_ is the energy of fentanyl, and ∑*E*_monomer_ is the total energy of the functional monomer.

## 3 Results and Discussion

### 3.1 In-Silico Screening of Functional Monomers

The formation of a stable template–monomer complex is critical to the success of MIP sensors. It is established that sturdier interaction between the functional monomer and template molecules will result in a more stable template–monomer complex prior to polymerization, consequently resulting better imprinting efficiency of the resulting polymer.^34^ The molecular interactions of five potential functional monomers with the target fentanyl were first studied via in-silico modeling. Geometry optimization of the complexes between fentanyl and the functional monomers was performed and the results are shown in Supporting Information Figure S1. The calculated negative binding energies between fentanyl and the monomers, shown in Figure 1, indicate that the system (complexes) formed between fentanyl and any of these monomers is stable, and the stability is enhanced with the number of functional monomers put around the template molecule. The plot also identifies dopamine as the best candidate functional monomer for the preparation of MIP toward fentanyl, and it was used in the preparation of the MIP sensor.

**Figure 1:**
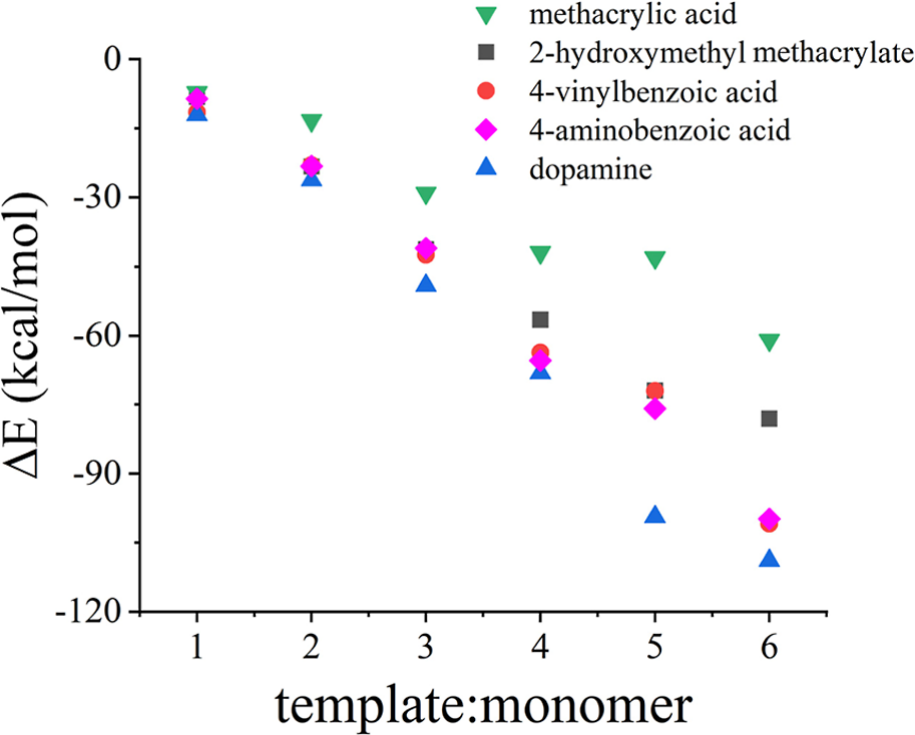
Interaction energy between fentanyl molecules and the different functional monomers.

### 3.2 Preparation and Characterization of the Fentanyl-Imprinted MIP

#### 3.2.1 Surface Modification of SPCE

The steps followed in the fabrication of the MIP fentanyl sensor are depicted in Scheme 1. First, the SPCE was modified with carboxylic acid-functionalized carbon nanofiber (fCNF) and Pt nanoparticles to increase the electrical conductivity and specific surface area of the electrode for the formation of MIP films. The functionalization of CNFs was characterized by FTIR, Raman spectroscopy, and zeta potential measurements. FTIR spectra of both as-received CNF and fCNF, Supporting Information Figure S2a, display intense peaks at 3469 cm^–1^, characteristic of an intermolecular OH stretching vibration; however, two new peaks at 1735 cm^–1^ and 1632 cm^–1^ were observed for fCNF that are ascribed to C=O stretching and OH vibration, respectively, indicating a successful functionalization process generating –COOH functional groups on CNF.^51^ The Raman spectra of both functionalized and as-received CNF, Supporting Information Figure S2b, reveal three major peaks centered at ∼1350 cm^–1^, ∼1590 cm^–1^, and ∼2690 cm^–1^ associated with the D, G, and 2D bands of CNF, respectively.^52^ These peaks delineate disorder and imperfection of the carbon crystallites.^52^ The ratio of the D-band and G-band intensity increased from 0.1 for CNF to 0.4 for fCNF, indicating the acid functionalization boosted the disorder.^52^ Zeta potentials of fCNF and as-received CNF, measured in water, were found to be −45.3 ± 0.6 and −24.1 ± 2.8 mV, respectively. The higher absolute value (more negative) zeta potential measured for fCNF signifies the presence of additional negative charges, which is attributed to the induction of COOH functional groups. EDS spectra of the fCNF/SPCE and fCNF-Pt/SPCE surfaces, shown as Supporting Information Figure S3, show predominantly carbon peaks and confirm the presence of Pt in the fCNF-Pt/SPCE electrode.

Supporting Information Figure S4a shows typical CVs of 1 mM [Fe­(CN)_6_]^3–/4–^ recorded at 0.1 V s^−1^ scan rate using bare SPCE, fCNF/SPCE, and fCNF-Pt/SPCE. The CV of a bare SPCE shows a quasi-reversible redox behavior with a peak-to-peak separation potential (Δ*E*_p_) of 0.22 V. Modification of the electrode with fCNF and fCNF-Pt decreased the Δ*E*_P_ value to 0.15 and 0.11 V, respectively, and increased the peak currents of the redox probe, indicating that the surface modification promoted charge transfer between [Fe­(CN)_6_]^3–/4–^ and the electrode. This observation is also supported by the drastic reduction in the charge-transfer resistance (*R*_ct_) of the fCNF-Pt/SPCE surface in EIS measurement (*R*_ct_ ∼3.6 kΩ for SPCE and <0.1 kΩ for fCNF-Pt/SPCE), as shown in Supporting Information Figure S4b. Supporting Information Figure S4c shows the peak currents (*i*_p_) of the redox species increased linearly with the square root of the scan rate (υ^1/2^) for all electrodes, suggesting a diffusion-limited redox process. Using the Randles–Sevcik equation^27^ to the data presented in Supporting Information Figure S4c, electroactive surface areas of 0.10 cm^2^, 0.13 cm^2^, and 0.17 cm^2^ were calculated for bare SPCE, fCNF/SPCE, and fCNF-Pt/SPCE, respectively. Similar electroactive surface area enhancements have been reported in the literature for CNF-modified SPCEs.^27,53^ The SEM images presented in Figure 2 (a–c) show a clear difference in the morphologies of the three electrodes, and the fCNFs were uniformly distributed and formed a 3D network structure. Figure 2c shows 10–35 nm Pt nanoparticles were deposited onto the fCNF/SPCE electrode, which enabled the fCNF-Pt/SPCE electrode to have a higher electroactive surface area and increased electrical conductivity.

**Figure 2:**
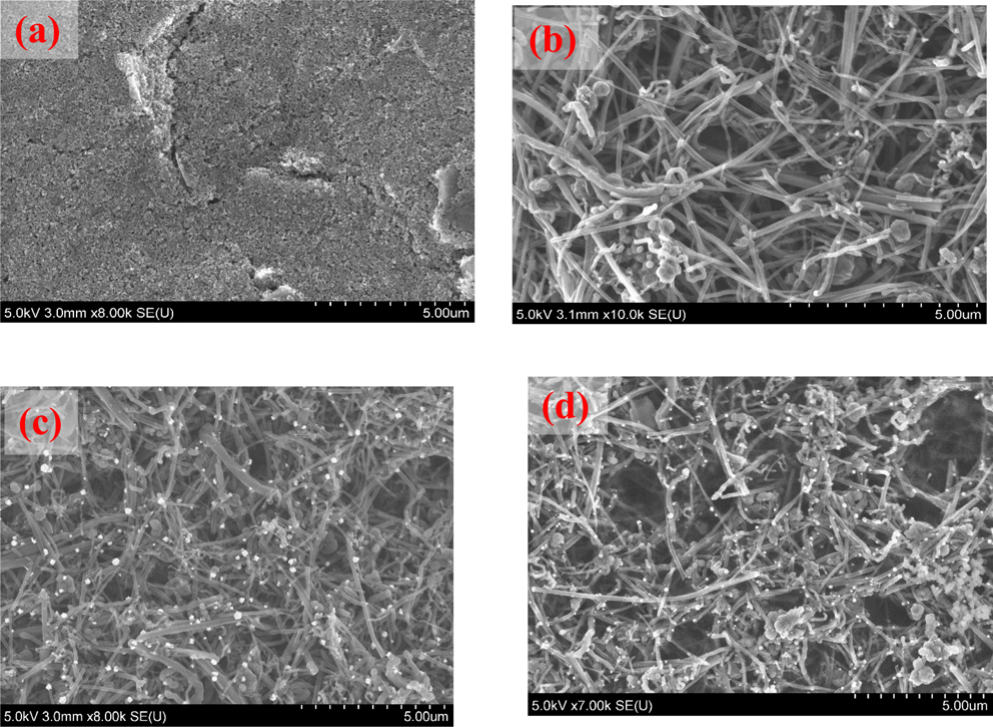
SEM images of (a) SPCE, (b) fCNF/SPCE, (c) fCNF-Pt/SPCE, (d) MIP/fCNF-Pt/SPCE.

### 3.3 Preparation and Characterization of MIP/FCNF–Pt/SPCE

Though dopamine undergoes slow autoxidation to polydopamine in oxygenated alkaline conditions,^38^ herein electropolymerization was employed to prepare MIP on the fCNF-Pt/SPCE electrodes as this process is fast, renders a higher deposition rate, and leads to complete and uniform polymer coverage on the electrode surface.^38,54^ The interaction between fCNF-Pt/SPCE and polydopamine MIP involves both physical and chemical interactions, including π–π stacking between the aromatic rings of dopamine and the graphitic structure of CNF, van der Waals forces, and electrostatic interactions. Figure 3a shows typical CV of the electropolymerization of dopamine on fCNF-Pt/SPCE in the presence and absence of fentanyl in the potential window of −0.5 V to +0.5 V (vs. Ag) using 0.1 M PB, pH 8.0. Two pairs of redox peaks (a1 and c1 pair at +0.15 V and +0.04 V, and a2 and c2 pair at −0.28 V and −0.40 V) were seen under both conditions except for the first cycle without a2, which have been attributed in the literature to redox couples of dopamine/ortho-dopaminoquinone (a1/c1) and leukodopaminechrome/dopaminechrome (a2/c2) generated due to the electropolymerization of dopamine.^38^ The oxidative polymerization of dopamine is proposed to follow a chemical reaction sandwiched between two electron transfer (ECE) mechanism, which eventually leads to a melamine-like polymer.^38^ As the electropolymerization cycle continues, the intensities of all the peak currents gradually decreased with their separation potentials between the redox pairs (a1/c1 and a2/c2) increased, indicating a continuous formation of an insulating polydopamine layer on the fCNF-Pt/SPCE surface as previously reported.^38,40^ No notable difference was observed in the CV of dopamine electropolymerization in the presence (MIP preparation) and absence (NIP preparation) of fentanyl, indicating that the template does not have any redox property in the potential window used in MIP preparation. This was confirmed in a separate experiment, Supporting Information Figure S5a, where the oxidation potential of fentanyl on fCNF-Pt/SPCE was determined to be ∼ +0.65 V (vs Ag). In the literature, the electrochemical oxidation of fentanyl is shown to be an irreversible process (Figure S5b) that proceeds through a two-electron coupled two-proton dealkylation of its tertiary amine resulting in norfentanyl and phenylacetaldehyde. From the voltammograms shown in Figures 3a and S5a, we concluded that the chemical structure of fentanyl is not affected by the imprinting process, which is important for the entrapment of intact fentanyl molecules, rather than the oxidation products (norfentanyl and phenylacetaldehyde). As discussed above, fentanyl is expected to interact with dopamine in the prepolymerization mixture via π–π interactions between the aromatic groups of the two compounds and hydrogen bonding between the carbonyl and tertiary amine groups of fentanyl and the hydrogen atoms of both the hydroxyl and amine groups of dopamine and get entrapped in the polymer. The cavities formed following removal of the entrapped template molecules from the MIP network are complementary to the size, shape, and position of fentanyl functional groups, which can lead to the selective recognition of analyte fentanyl molecules.^55^

**Figure 3:**
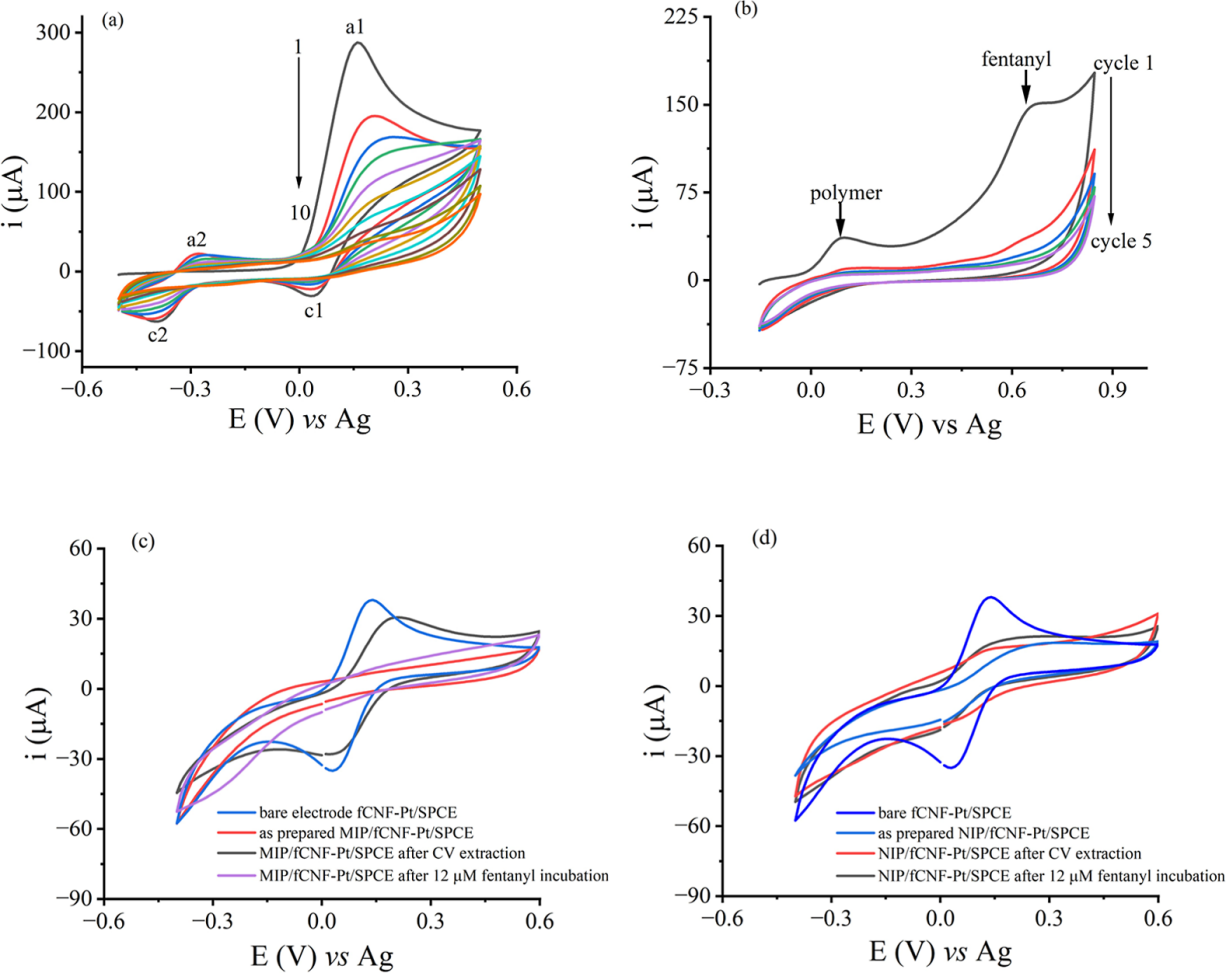
CVs of (a) the electropolymerization of a mixture of dopamine and fentanyl (1:6 molar ratio) on fCNF-Pt/SPCE at a scan rate of 50 mV/s. (b) The electrochemical extraction of entrapped fentanyl. (c) and (d) show the behavior of 1 mM [Fe­(CN)^6^]^3–/4–^ on the as-prepared MIP and NIP surfaces and following the extraction and incubation of fentanyl (12 μM) at a scan rate of 100 mV/s. All experiments were obtained using 0.1 M PB, pH 8.0, as an electrolyte.

Following MIP and NIP preparations on fCNF-Pt/SPCEs, the behavior of the resulting surfaces was characterized by CV using [Fe­(CN)_6_]^3–/4–^. As seen in Figure 3c,d, after electropolymerization and before template removal, the redox peaks of [Fe­(CN)_6_]^3–/4–^ were significantly reduced for both MIP- and NIP-modified electrodes, demonstrating successful polymerization of a nonconductive polydopamine layer. Following a subsequent step of template removal, the peak currents of the redox probe on the MIP/fCNF-Pt/SPCE surface increased to ∼82% of the bare electrode currents but not on the NIP/fCNF-Pt/SPCE surface. These observations demonstrate successful formation of cavities on the MIP network that facilitated the transfer of electrons between the redox probe and the electrode surface. Following incubation of the template-extracted MIP/fCNF-Pt/SPCE sensor with 12 μM fentanyl, the CV of the sensor showed a major reduction in the redox probe’s current intensity, indicating that a significant portion of the cavities formed in the MIP film were filled with fentanyl molecules blocking the redox probe from approaching the electrode surface. The CVs of the NIP/fCNF-Pt/SPCE remained unchanged following fentanyl extraction and incubation with 12 μM fentanyl, Figure 3d.

Comparing the SEM images shown in Figure 2c,d, the size of the fCNF-Pt network structure remarkably increased following the preparation of MIP on the electrode surface. Also, the Pt nanoparticles that were evident in fCNF-Pt/SPCE (Figure 2c) were covered with a polymer layer following electrodeposition of the MIP (Figure 2d), providing further evidence to the electropolymerization of dopamine on the fCNF-Pt/SPCE surface.

### 3.4 Optimization of the MIP Sensor Preparation

To design a sensor with an optimal analytical performance, parameters such as the number of CV polymerization scan cycles, the molar ratio of template to monomer molecules, pH of the electropolymerization supporting electrolyte, template extraction conditions, and incubation time were investigated and analyzed. The resulting data are presented in Supporting Information Figure S6.

#### 3.4.1 Electropolymerization Scan Cycle

The number of electropolymerization cycles affects the thickness of the polymer layer, which in turn affects the sensor’s performance.^38^ Hence, a prepolymerization solution composed of a 1:8 molar ratio of fentanyl:dopamine (0.35 mM fentanyl and 2.8 mM dopamine, in 0.1 M PB pH 7.4) was electropolymerized on different fCNF-Pt/SPCEs by varying the CV cycle number from 5 to 30. DPVs of fentanyl in the resulting polymerized surfaces (MIP/fCNF-Pt/SPCEs) were recorded in 0.1 M PB, pH 7.4, and the resulting current densities are presented in Supporting Information Figure S6a. The plot shows 10 cycles of polymerization-yielded maximum response. Further increasing the electropolymerization scan cycles resulted in decreased fentanyl oxidation current, which could be due to the formation of a thick MIP layer impeding the oxidation of fentanyl molecules entrapped near the polymer surface. Thick polymer films could also result in insufficient recognition sites and prolong drug extraction and incubation times.^38^ Thus, 10 cycles of polymerization were chosen as optimal for the sensor fabrication.

#### 3.4.2 Molar Ratio of the Template to Monomer

The relative amount of template molecules to functional monomers in the prepolymerization mixture affects the number of imprinted cavities and the thickness of the MIP film, which in turn affects the sensor’s performance.^38^ Prepolymerization mixtures composed of different fentanyl-to-dopamine molar ratios (1:4, 1:6, 1:8, and 1:10) were prepared and electrochemically polymerized onto various fCNF-Pt/SPCEs. Data presented in Supporting Information Figure S6b depicts the DPV signal of fentanyl reached a maximum at a 1:6 molar ratio. Further increasing the monomer concentration resulted in a decreased fentanyl signal, which is ascribed to the excess amount of the monomer resulting in an increased MIP film thickness and a resultant reduction in the conductivity of the MIP film. Thus, a fentanyl-to-dopamine molar ratio of 1:6 was set for further experiments.

#### 3.4.3 pH of Electropolymerization and Incubation Solutions

The interaction between the functional monomer and the template molecule as well as the structural stability of the imprinted polymer are impacted by the pH of the electropolymerization solution.^38^ Previous literature reports on dopamine-based MIPs show optimal electrochemical sensors’ performance at pHs of 7.5 to 8.0.^38^ Our group previously reported that the oxidation of fentanyl on fCNF/SPCE is pH dependent and optimal response was obtained at pH 8.0.^27^ Here, different prepolymerization solutions consisting of a 1:6 molar ratio of fentanyl-to-dopamine were prepared in 0.1 M PB at different pHs (6, 7.4, 8, and 9) and the DPV signals of the resulting MIPs were recorded and compared. Supporting Information Figure S6c shows that sensors prepared at pH 7.4 and 8 yielded maximum current for fentanyl, and pH 8 was chosen for sensor fabrication. Similarly, we optimized the pH of the fentanyl incubation solution on the rebinding of fentanyl to the MIP sensor by incubating with 10 μM fentanyl prepared in 0.1 M PB at varying pHs. Supporting Information Figure S6d illustrates that pH 8 gave a slightly higher fentanyl DPV current and was selected for further studies.

#### 3.4.4 Extraction Condition and Incubation Time

Following the formation of an MIP film on the electrode, efficient and complete removal of template molecules from the polymer network is the most crucial step. Incomplete removal of template molecules from an MIP surface can result in fewer cavities, thereby reducing sensor efficiency. Solvent extraction methods, which are based on the selective solubility of template molecules in a solvent in which the polymer network is insoluble, are the common approaches for template removal in MIP preparation.^38,54,56^ However, these methods require trial-and-error-based choice of organic (sometimes organic and aqueous mixture) solvents and long extraction times and can lead to incomplete removal of template molecules and damage the imprinted cavities.^56^ For example, Li et al.^32^ determined incubating a fentanyl–polyarginine MIP sensor with a mixture of methanol and acetic acid (9:1, v/v) for 6 min removed most of the entrapped fentanyl template. In this work, we exploited the electrochemical oxidation of fentanyl to remove the entrapped drug from an MIP formation and create cavities. Figure 3b depicts CVs of an as-prepared MIP run in 0.1 M PB pH 8.0 buffer. The complete disappearance of the fentanyl oxidation peak in subsequent CV cycles (peak was observed in the first CV cycle at a peak potential of ∼0.65 V) indicates successful removal of the entrapped fentanyl molecules from the MIP network. Thus, the electrochemical method, which is fast and efficient, was used in this study.

To investigate the relationship between the MIP sensor electrochemical response and the rebinding time of the drug, 100 μL of 10 μM fentanyl (prepared in 0.1 M PB, pH 8.0) was incubated with the sensor for varying lengths of time. Supporting Information Figure S6e shows that the sensor response gradually increased, reaching a maximum at 15 min. Consequently, an incubation time of 15 min was chosen for further studies.

### 3.5 Analytical Performance of the MIP Sensor

The analytical performance of the imprinted sensor toward the determination of fentanyl was studied by a DPV technique. After template removal, imprinted sensors were incubated with varying concentrations of fentanyl for 15 min. Figure 4a shows the oxidation current of fentanyl increases with increasing its concentration, implying that more imprinted cavities in the sensor were occupied with the drug. From the figure, it is evident that the oxidation of fentanyl yields two oxidation peaks, labeled ox1 and ox2 recorded at peak potentials of ∼0.65 V and ∼0.73 V, respectively. This observation is consistent with the literature-reported electrochemical oxidation of fentanyl on carbon electrodes.^24,26,27,57^ In Figure 4a, the ox2 peak can no longer be clearly observed at concentrations less than 8 μM, thus the calibration plot depicted in Figure 4b was generated using the current intensity of ox1. The plot shows saturation behavior at higher concentrations of fentanyl, suggesting the binding of fentanyl to the MIP sensor transpires according to the Langmuir adsorption model. This was corroborated by a good theoretical fit to the Langmuir–Freundlich isotherm equation,^58^ with a correlation coefficient (*R* ^2^) of 0.9987. From the fit, we calculated the maximum achievable saturation current to be 11.04 μA, and the adsorption capacity of the MIP sensor (i.e., dissociation constant, *K*_d_) is 16.13 μM. A calculated heterogeneity factor of 0.7 implies that the binding cavities across the MIP network have different energies (i.e., different affinities for fentanyl), and adsorption sites with lower energy are more prevalent and the adsorption process is more favorable at lower concentrations but less so as the fentanyl concentration increases.^58^ The limit of detection (LOD) of the sensor, calculated as 3σ_intercept_/slope (σ is the standard deviation) from the parameters of a linear fit (*i* = 0.554 [fentanyl] + 9.95 × 10^–5^, *R* ^2^ = 0.9894) to the lowest concentration range (0.5–10 μM), was found to be 0.094 μM. As shown in Table 1, while this LOD is better than or comparable to most literature-reported values for bare carbon-based fentanyl sensors,^19,21–27^ it is higher than the only literature-reported value for an MIP-based fentanyl electrochemical sensor (LOD 1.2 nM, with a dynamic range of up to 1.7 μM).^32^ Compared to our sensor, the narrow dynamic range reported in Li et al.’s^32^ MIP sensor might be due to the very high potential required to electropolymerize l-arginine (CV was conducted in the range of −2.0 V to +2.0 V by Li et al.,^32^ a potential window where fentanyl undergoes electro-oxidation). The sensitivity of our sensor (580 μA/mM) is also slightly better than the one reported by Li et al. (370 μA/mM).^32^ As noted above, to prepare efficient MIP electrochemical sensors for electroactive substances, effective monomers should undergo polymerization at potentials below the oxidation or reduction potentials of the analyte. The use of dummy templates (i.e., structural analogues) is also a common practice employed to enhance the performance of electrochemical MIPs when the target analyte is unstable in the polymerization potential window.^59^

**Figure 4:**
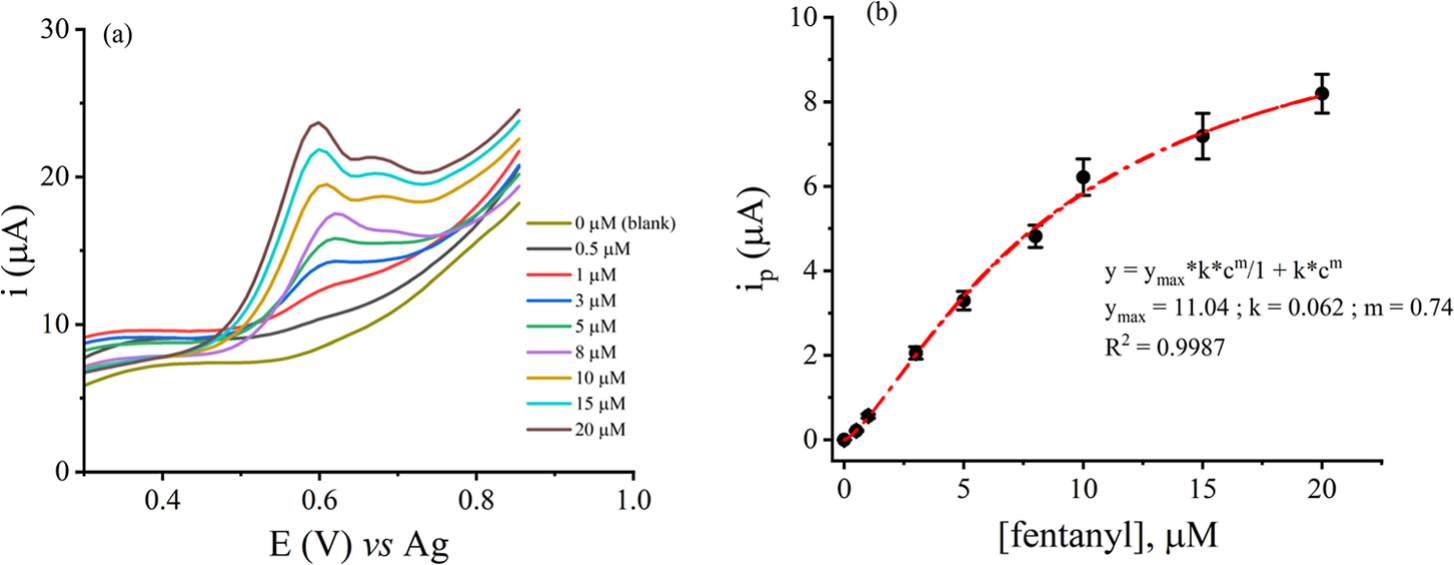
(a) DPV of different concentrations of fentanyl obtained using MIP/fCNF-Pt/SPCE. (b) Calibration plot generated from data in (a). Each data point was generated from 3 independent MIP sensors prepared and processed in parallel, and the error bars represent standard deviations. The red line in (b) represents the best fit for a nonlinear regression of heterogeneous binding sites using the Langmuir–Freundlich isotherm.

**Table 1:** Comparative Analytical Performance of the MIP/fCNF-Pt/SPCE Sensor with Literature-Reported Fentanyl Electrochemical Sensors^a^

sensor	method	matrix	linear range (μM)	LOD (μM)	reference
NiO-AuNp/SPCE	LSV	0.1 M PB, pH 7.0	0.25–25	0.1	^18^
MWCNT and ionic liquid/SPCE	SWV	0.1 M PB, pH 7.4	10–100	10	^19^
MWCNT/GCE	DPAdSV	0.1 M PB, pH 7.4	0.5–100	0.1	^23^
SPCE	SWAdSV	0.1 M Tris–HCl, pH 8.5	0.23–20.5	0.69	^24^
carbon nano-onion/GCE	DPV	0.1 M PB, pH 7.4	1–60	0.3	^25^
fCNF/SPCE	DPV	0.1 M PB, pH 8.0	0.125–10	0.075	^27^
SIMPLE-3D-SenS	SWV	0.15 M BR buffer, pH 6.5	0.1–9.1	0.017	^29^
ZIF-8-MOF/SPGE	SWV	0.1 M PBS, pH 7.0	0.1–1	0.03	^31^
MIP/rGO/GCE	SWV	0.1 M PB, pH 7.4	0.04–1.72	0.013	^32^
ionic liquid/SPCE	SWV	0.1 M PB, pH 7.4	10–100	5	^57^
MIP/fCNF-Pt/SPCE	DPV	0.1 M PB, pH 8.0	0.5−10	0.094	this work

a MOF = metal organic framework, ZIF-8-MOF = zeolite imidazolate framework-8, GCE = glassy carbon electrode, LSV = linear sweep voltammetry, DPAdSV = differential pulse adsorptive stripping voltammetry, SWAdSV = square-wave adsorptive stripping voltammetry, rGO = reduced graphene oxide, NP = nanoparticle, PBS = phosphate buffer saline, BR = Britton–Robinson buffer, SWV = square-wave voltammetry, SIMPLE-3D-Sens = 3D-printed conductive carbon black electrode.

The selectivity of the MIP sensor toward fentanyl was assessed by spiking equimolar concentrations of some of the common street drug-cutting agents (glucose, acetaminophen, theophylline) and other opioids (morphine and codeine) as well as to norfentanyl (the oxidation product of fentanyl) and the opioid-antidote, naloxone, into 5 μM fentanyl solution. Figure 5 shows the fentanyl current responses of the MIP sensors were not affected by the presence of these chemicals, demonstrating the sensor discriminating ability. The selectivity coefficients (*k* = *i*_fentanyl_/*i*_interfering compound_, where *i* is the DPV current) of the developed sensor, determined from individually run 5 μM fentanyl and the interfering compounds shown in Figure 5, ranged from 11.6 for morphine to 187.9 for acetaminophen, indicating that the MIP sensor is more selective toward fentanyl than the interfering compounds. This compares favorably to our earlier report of using bare fCNF/SPCE for electrochemical determination of fentanyl,^27^ where the DPV signal for acetaminophen was observed at a lower potential than that of fentanyl, while signals for theophylline overlapped to that of fentanyl.^27^ Codeine and morphine also showed similar peak oxidation potentials as fentanyl on bare fCNF-Pt/SPCE (data not shown). For all analytes, the signal from the control NIP accounted for ≤18% of that of the MIP response. The selectivity of the MIP sensor toward fentanyl is ascribed to discrete molecular interactions between the fentanyl functional groups and the preformed cavities in the imprinted polymer network which match the structure of fentanyl sterically.

**Figure 5:**
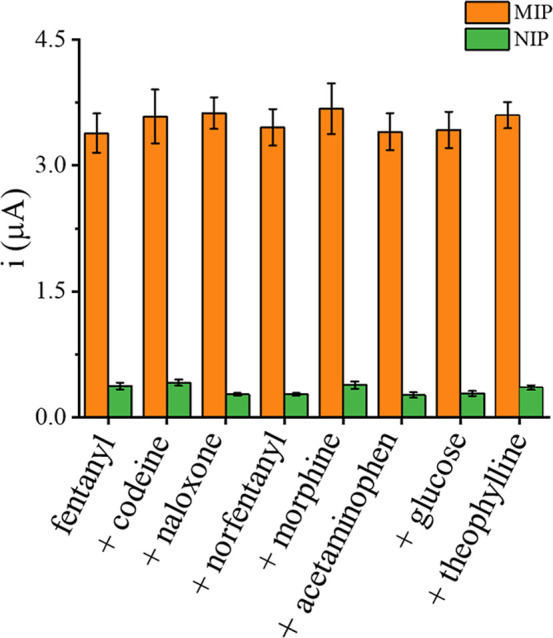
DPV response of MIP- and NIP-modified fCNF-Pt/SPCEs toward 5 μM of fentanyl spiked with equimolar concentrations of different compounds.

The electrochemical performance of an MIP sensor was evaluated by taking 5 replicate DPV measurements of 5 μM fentanyl on the same day, yielding a relative standard deviation of 6.7%. Comparing the SEM images (Supporting Information Figure S7) and EDS data (Supporting Information Figure S3) of the freshly prepared MIP/fCNF-Pt/SPCE sensor and the sensor after 5 replicate measurements showed no noticeable differences, indicating the sensor’s structure was not impacted during repeated measurements. The storage stability of the sensor was also assessed by periodically measuring the DPV responses of a batch of three MIP/fCNF-Pt/SPCE sensors to 5 μM fentanyl at various intervals over 4 weeks. Between tests, the sensors were rinsed and stored in DI water in a humidified environment at 4 °C. As illustrated in Figure 6, the sensor’s responses taken at seven distinct time points over the 4 week period showed little deviation (<10%) from their original responses, with average DPV currents (*n* = 7) of 3.52 ± 0.27 μA, 3.26 ± 0.22 μA, and 3.32 ± 0.24 μA for MIP 1, MIP 2, and MIP 3, respectively. The tight RSD (<7.7%) of the sensors indicates the MIP sensors are robust and stable with repeated storage and test cycles.

**Figure 6:**
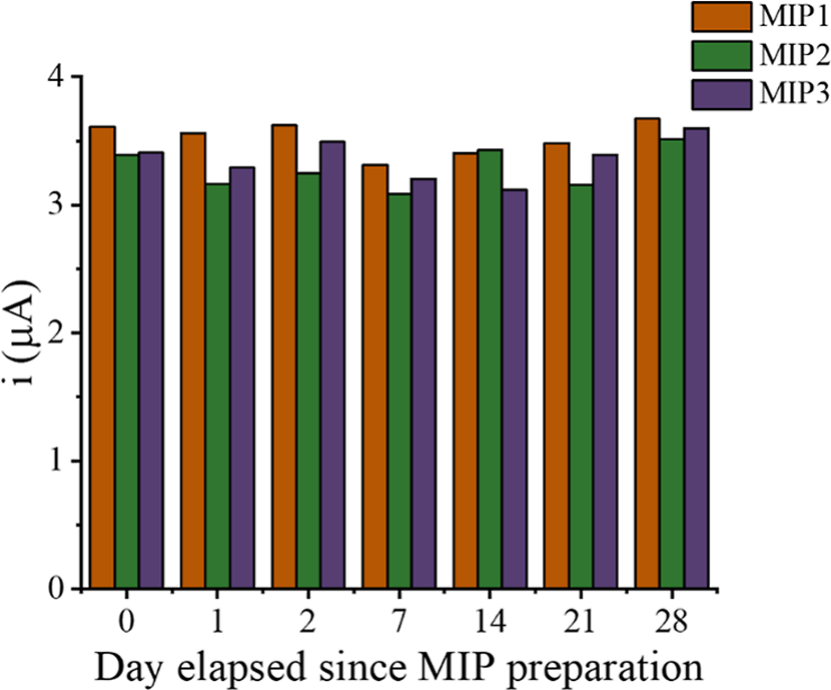
DPV responses of three MIP/fCNF-Pt/SPCE sensors, prepared and processed in parallel, in response to 5 μM fentanyl over a period of 28 days. After each measurement, sensors were rinsed and stored in water under a humidified environment at 4 °C.

To demonstrate the applicability of the developed MIP sensor in real-sample analysis, synthetic urine samples (composition shown in Supporting Information Table S1) were diluted 1:10 (v/v) in 0.1 M PB, pH 8.0 and spiked with different concentrations of fentanyl. Figure 7 shows analysis of the resulting fentanyl DPV signals, obtained from three independently prepared MIP sensors, followed a Langmuir–Freundlich adsorption isotherm (*R* ^2^ = 0.9955) in the studied concentration range of 0–20 μM fentanyl. The sensor displayed a linear relationship for 2–10 μM fentanyl in the urine sample (*i* = 0.381 [fentanyl] + 0.407; *R* ^2^ = 0.9829), with a calculated LOD of 0.9 μM. The higher detection limit and lower sensitivity obtained in spiked urine samples compared to that in buffer (Figure 4b) suggest that the urine matrix has an interfering effect in the performance of the MIP fentanyl sensor. Though no peaks other than the fentanyl oxidation peak were recorded during DPV, the reduced performance of the sensor implies chemicals in the urine sample nonspecifically interacted with the binding cavities of the MIP, compromising the number of available cavities for fentanyl interaction, or adsorbed onto the exposed fCNF-Pt/SPCE, poisoning the surface. Recently, several strategies have been suggested in the literature to minimize nonspecific adsorption of matrix components when using MIP sensors in real environmental and biological samples, including electrostatic modification of MIP surfaces with surfactants, building hydration layers using zwitterionic polymers or peptides, and using inert cladding to cover the MIP surface.^60^ Such strategies could be implemented in the future to enhance the performance of our fentanyl electrochemical sensor when analyzing real biological samples, such as urine. Nonetheless, the developed sensor responded to micromolar concentrations of fentanyl in the presence of interfering compounds in urine samples. The plot also shows the NIP did not have appreciable response to fentanyl in spiked urine samples.

**Figure 7:**
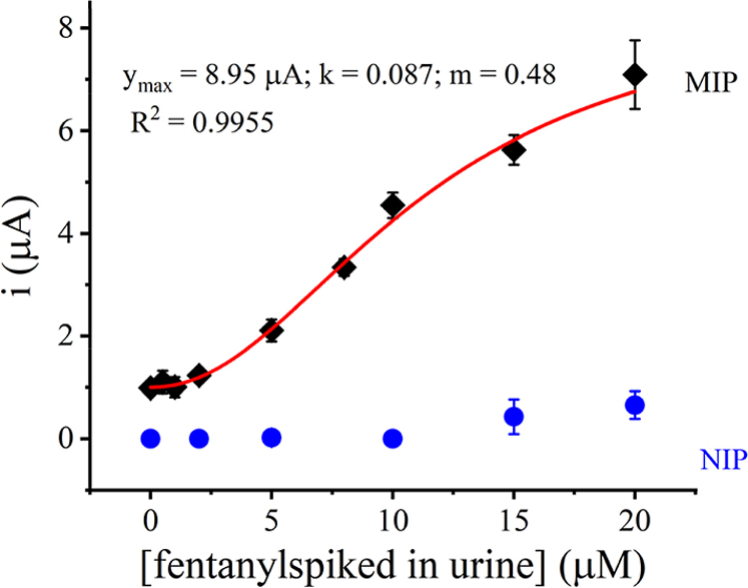
Calibration plot showing the DPV current response of MIP/fCNF-Pt/SPCE interrogated with fentanyl-spiked artificial urine samples. The red line represents the best fit for a nonlinear regression of heterogeneous binding sites using the Langmuir–Freundlich isotherm. The blue circles show the response of NIP/fCNF-Pt/SPCE to varying concentrations of fentanyl-spiked urine samples. Each data point was generated from 3 independent MIP and NIP sensors prepared and processed in parallel, and the error bars represent standard deviations.

## 4 Conclusion

This report demonstrates the development of an MIP-based electrochemical fentanyl sensor by electropolymerizing dopamine, the functional monomer, on the surface of fCNF-Pt/SPCEs. The developed sensor yielded a detection limit of 94 nM fentanyl, good run-to-run measurement, batch-to-batch fabrication repeatability, and excellent storage stability for at least 4 weeks when stored in a humidified environment in a fridge. The sensor also demonstrated excellent selectivity against other opioids and common cutting agents found in street drugs, and it can detect μM concentrations of fentanyl in urine-spiked samples. The combination of MIP with electrochemical sensing provides a sensitive and rapid approach for the determination of fentanyl, which could be particularly useful for applications in drug enforcement and health care settings where quick and reliable fentanyl detection is crucial.

## Supplementary Material



## Notes

Additional experimental details showing the interaction of fentanyl to the five modeled functional monomers, characterizing the functionalized carbon nanofibers via FTIR and Raman spectroscopy, EDS, SEM, and electrochemical characterization of the various stages of the sensor fabrication, electrochemical oxidation mechanism of fentanyl, MIP sensor performance parameter optimization, and composition of artificial urine used (PDF)
