# Supplementary material for: A Polydopamine-Based Molecularly Imprinted Electrochemical Sensor for Fentanyl Determination
Source: ACS Omega. 2025 Aug 12;10(33):38292–302. doi: 10.1021/acsomega.5c06732 (PMC12392191; doi:10.1021/acsomega.5c06732)
Supplement: Supplementary file 1 [file ao5c06732_si_001.pdf]

Supplementary material

A polydopamine-based molecularly imprinted electrochemical sensor for fentanyl determination

Michelle Tong[1], Rajesh Pillai[1], Alexander Kobryn[1], Zhimin Yan[1], Nora W. C. Chan[2,3] and Abebaw B. Jemere[1,3]*

1 National Research Council Canada – Quantum and Nanotechnologies Research Centre, Edmonton, AB, T6G 2M9, Canada
2Defence Research and Development Canada - Suffield Research Centre, Medicine Hat, AB, T1A 8K6, Canada
3Department of Chemistry, Queen's University, Kingston, ON, K7L 3N6, Canada

*Corresponding author: abebaw.jemere@nrc-cnrc.gc.ca

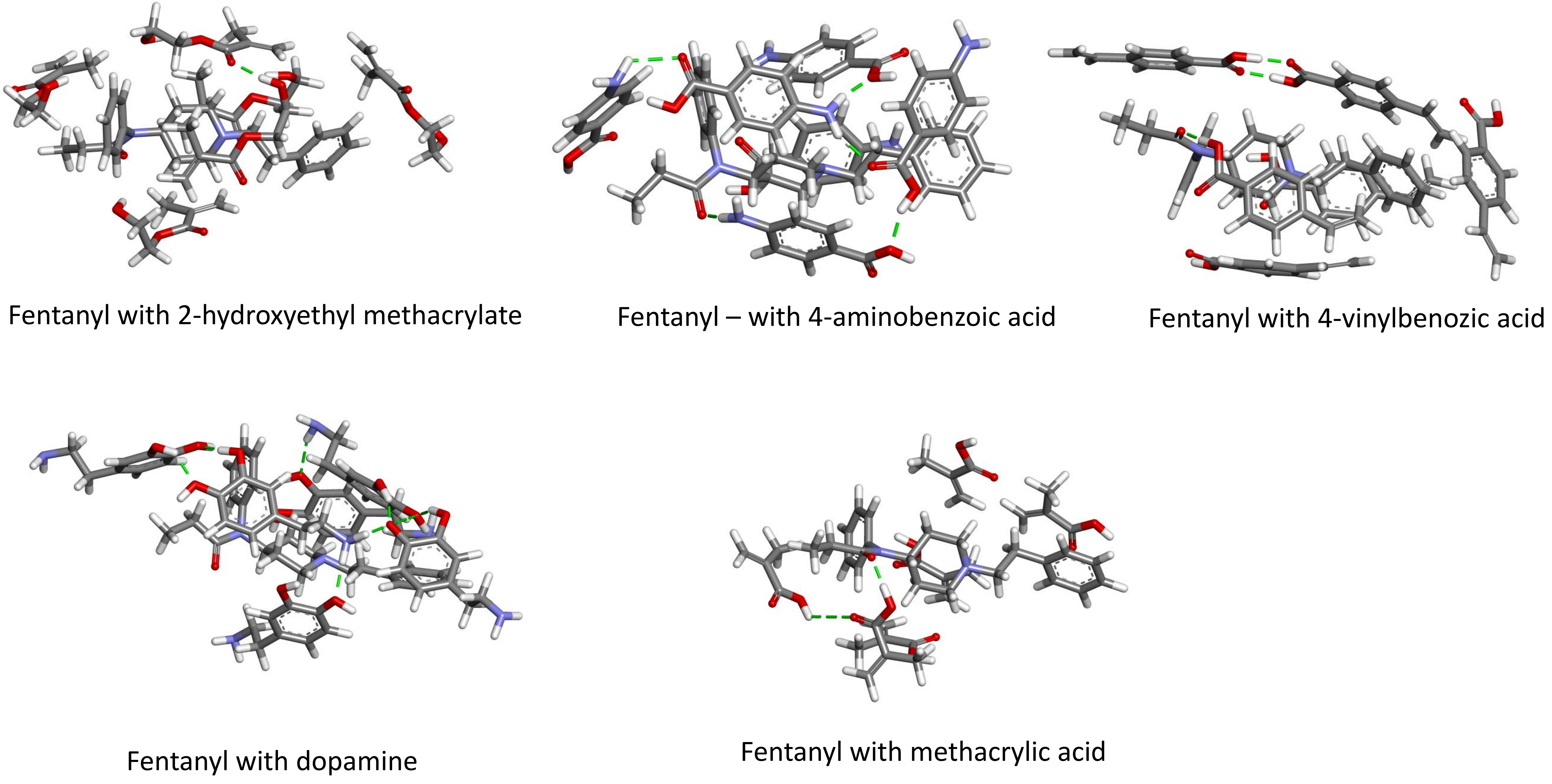

Supplementary Figure S1. In silico modeling showing the interaction of five monomers with fentanyl. Green lines shows hydrogen bonding.

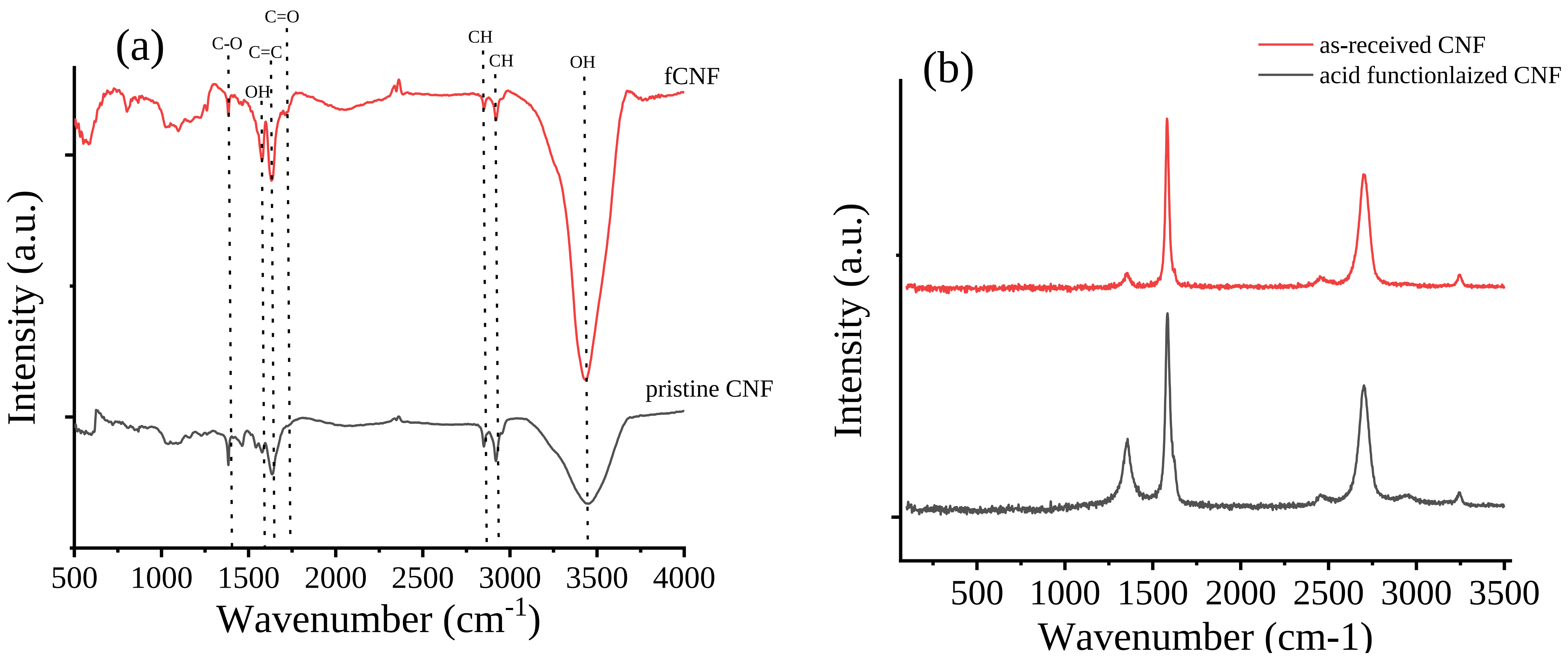

Supplementary Figure S2. (a) FTIR and (b) Raman spectra of as-received and acid functionalized carbon nanofiber.

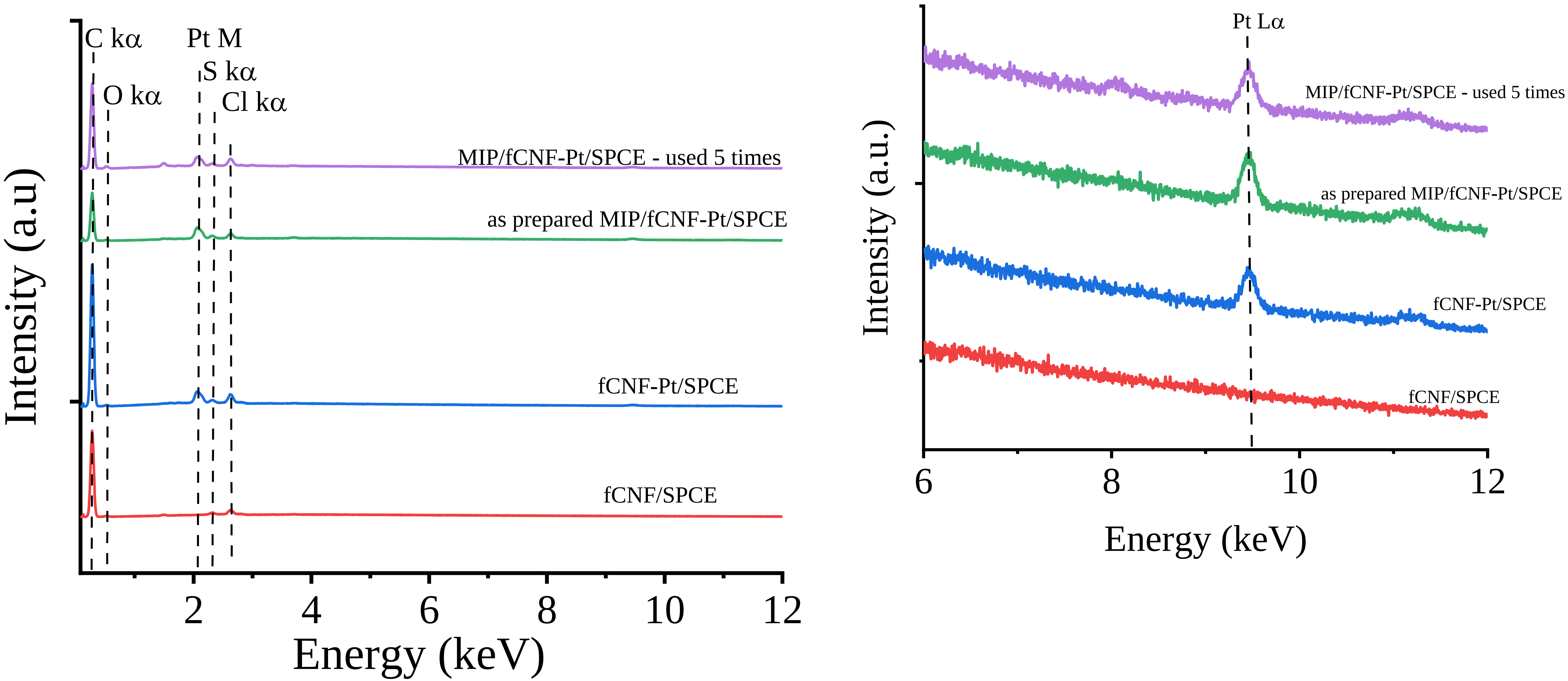

Supplementary Figure S3 . EDS spectra of the electrodes at various stages of preparation. An MIP modified electrode was analyzed right after preparation and following five repetitive fentanyl incubation and detections. The inset shows an expanded view of the region between 6 and 12 keV.

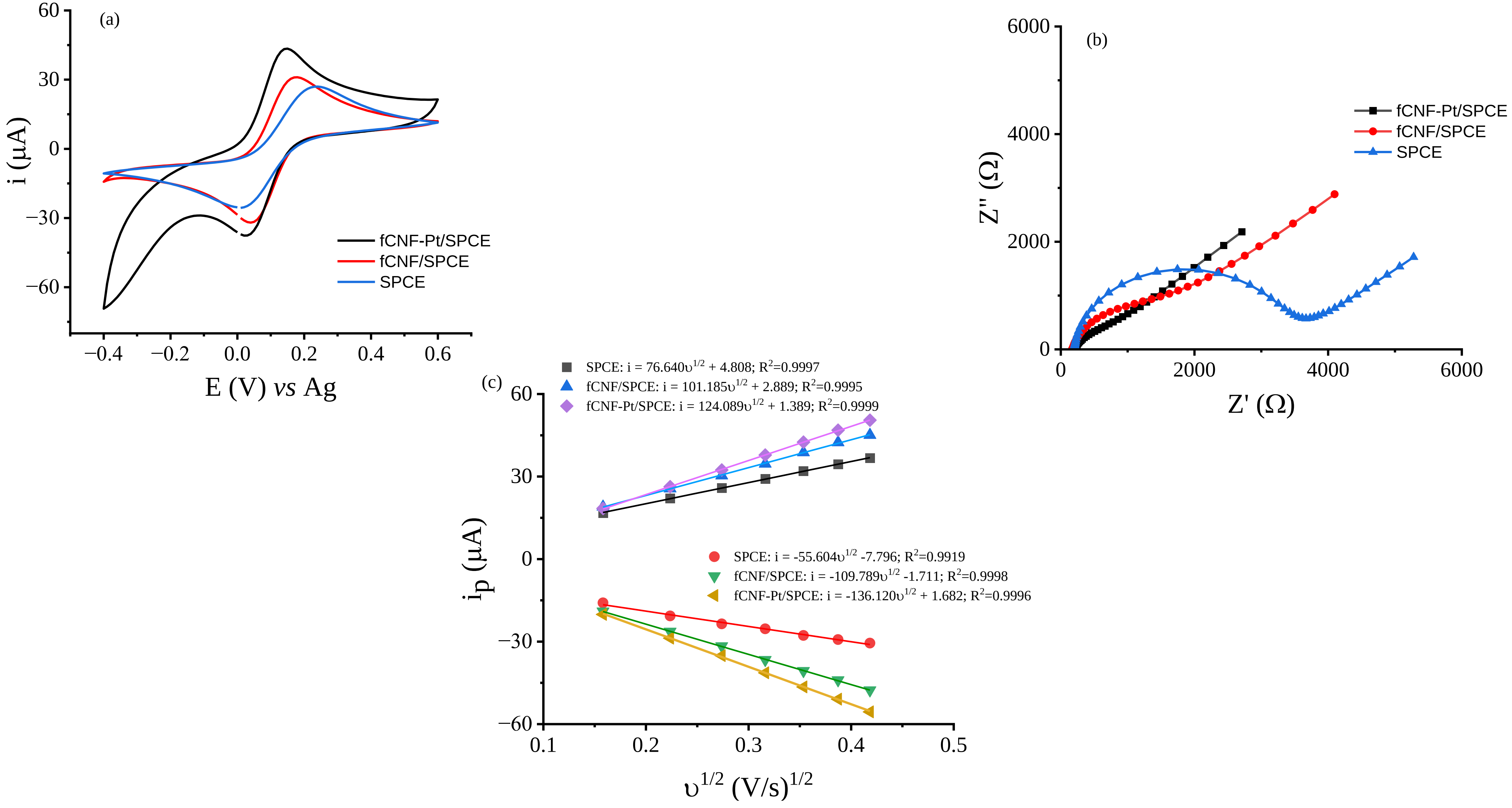

Supplementary Figure S4. Typical CV (a) and EIS (b) of 1 mM $[Fe(CN)_6]^{3-/4-}$ obtained using bare and modified SPCE at a scan rate of 100 mV/s. (c) shows the plot of peak cathodic and anodic currents of the redox probe vs SQRT of various scan rates obtained using the three electrodes.

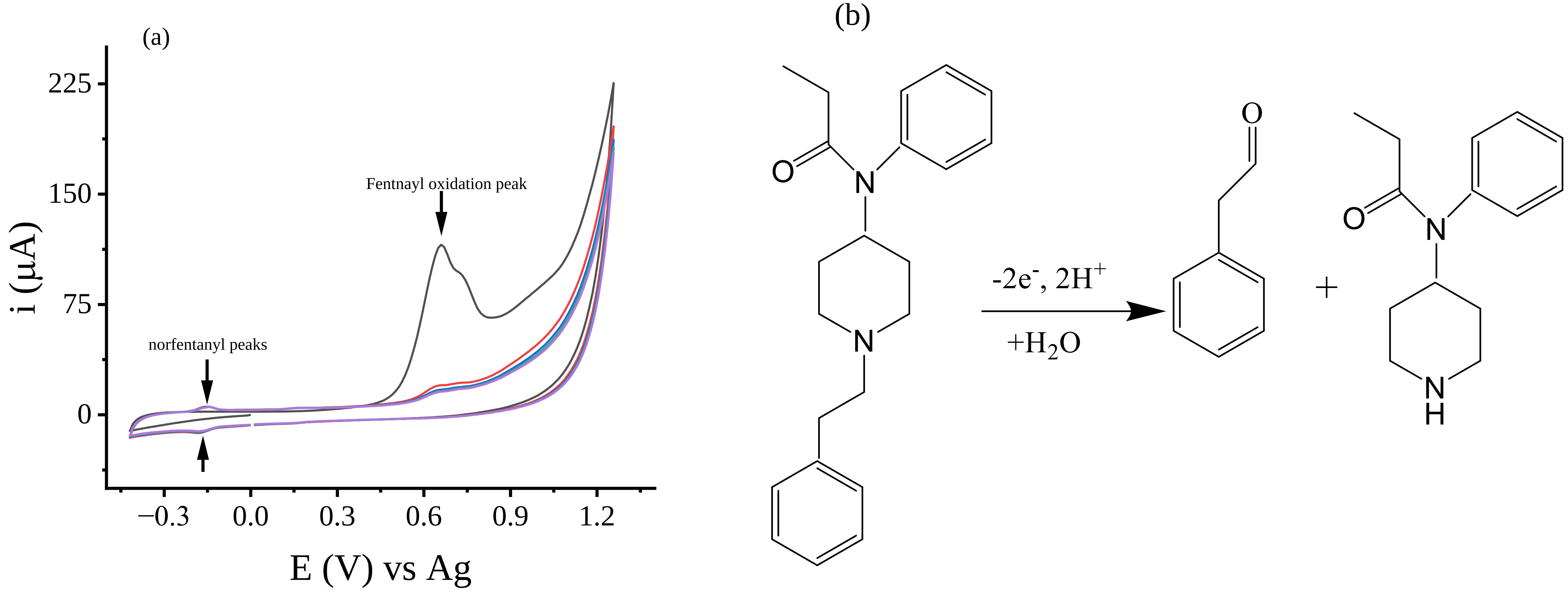

Supplementary Figure S5. (a) CV of the electrooxidation of 100 μM fentanyl on a fCNF-Pt/SPCE using 0.1M phosphate buffer, pH 8.0. The redox peak pairs of the oxidation product of fentanyl (i.e. norfentanyl) are also marked. The oxidation peak of fentanyl significantly decreased after the first cycle, indicating the surface's catalytic power; and (b) electrooxidation mechanism of fentanyl to norfentnayl and phenylacetaldehyde.

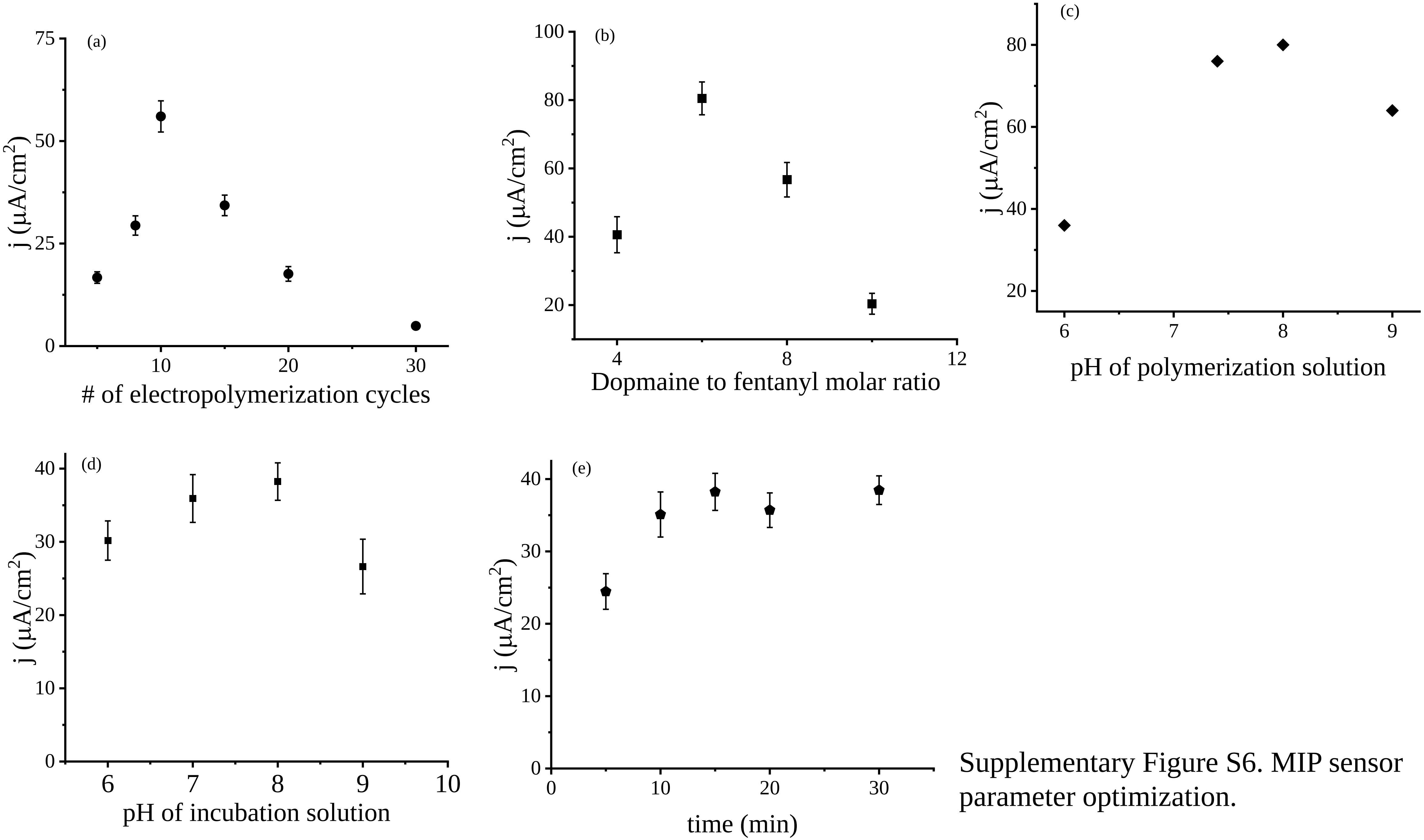

Supplementary Figure S6. MIP sensor parameter optimization.

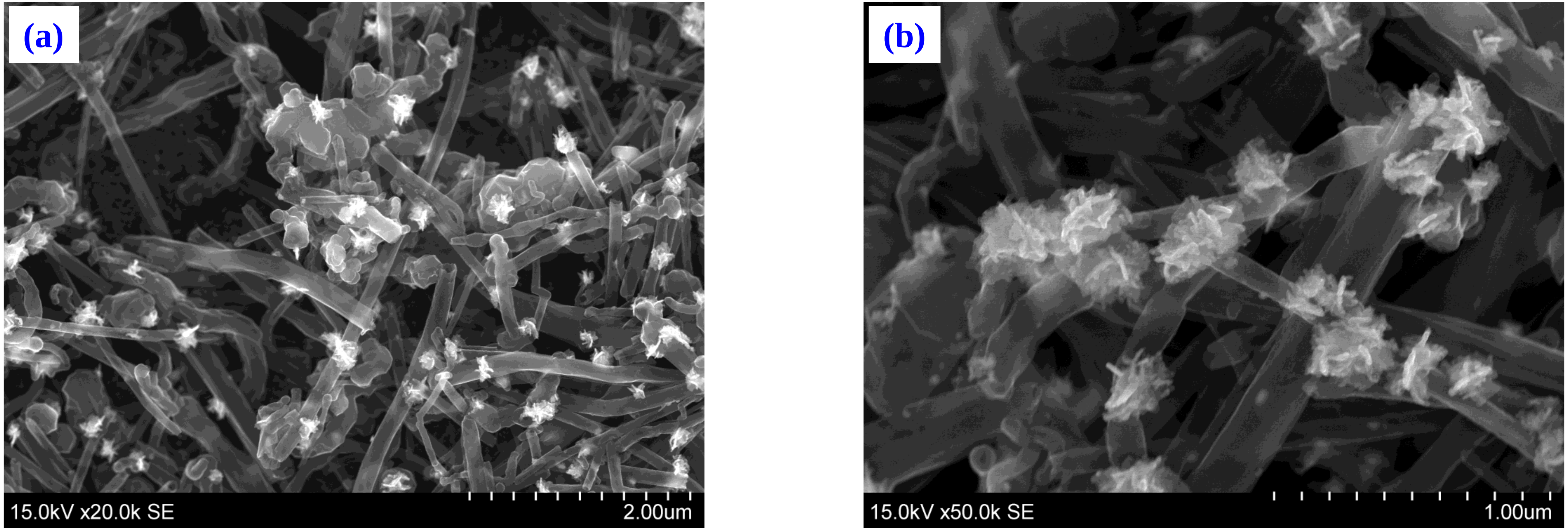

Figure S7. SEM images of (a) freshly prepared and (b) 5 times used MIP/fCNF-Pt/SPCE sensor.

Supplementary Table S1. Composition of artificial urine

**Ingredient**	**Concentration (g/L)**
Urea	25
Sodium chloride	9
Disodium hydrogen orthophosphate, anhydrous	2.5
Potassium dihydrogen orthophosphate	2.5
Ammonium chloride	3
Creatinine	2
Sodium sulphite, hydrated	3
